# Early Enteral Feeding After Living Donor Liver Transplantation Prevents Infectious Complications: A Prospective Pilot Study

**DOI:** 10.1097/MD.0000000000001771

**Published:** 2015-11-06

**Authors:** Jong Man Kim, Jae-Won Joh, Hyun Jung Kim, Sung-Hye Kim, Miyong Rha, Dong Hyun Sinn, Gyu-Seong Choi, Choon Hyuck David Kwon, Young Yun Cho, Jeong-Meen Suh, Suk-Koo Lee

**Affiliations:** From the Department of Surgery, Sungkyunkwan University School of Medicine (JMK, J-WJ, HJK, G-SC, CHDK, J-MS, S-KL); Department of Dietetics (S-HK, MR, YYC); and Division of Gastroenteology, Department of Medicine, Sungkyunkwan University School of Medicine, Seoul, South Korea (DHS).

## Abstract

Infectious complications, including bacteria, virus, and fungus, often occur after liver transplantation and are the most frequent causes of in-hospital mortality. The current study prospectively analyze the effect of early enteral feeding in patients after living donor liver transplantation (LDLT)

Between January 2013 and August 2013, 36 patients underwent LDLT. These patients were randomly assigned to receive enteral formula via nasointestinal feeding tubes [enteral feeding (EN) group, n = 17] or maintenance on intravenous fluid until oral diets were initiated (control group, n = 19). All patients completed the study.

The pretransplant and perioperative characteristics of patients did not differ between the 2 groups. The incidence of bacterial infection was significantly lower in the EN group (29.4%) than in the control group (63.2%) (*P* = 0.043). In addition, the incidence of bile duct complications in the EN group was lower than in the control group (5.9% versus 31.6%, *P* = 0.041). Multivariate analysis showed that early enteral feeding was closely associated with bacterial infections (odds ratio, 0.178; *P* = 0.041). There was no statistically significant difference in nutritional status between the 2 groups. There were no cases of in-hospital mortality.

Early enteral feeding after LDLT prevents posttransplant bacterial infection, suggesting the possibility of a reduction of in-hospital mortality as a result of decreased infectious complications.

## INTRODUCTION

Nutrient metabolism is changed in patients with liver disease because liver is a central organ for metabolism. Thus, protein malnutrition and imbalance were developed as a result of progressive liver disease.^1–4^ Protein-energy malnutrition is common in patients with end-stage liver disease requiring liver transplantation (LT). Recently, only the sickest patients with the highest model for end-stage liver disease scores have received transplants because organs from deceased donors are relatively scarce. These patients, who are significantly malnourished and physically deconditioned, have an increased risk of posttransplant morbidity and mortality.^5,6^

Infectious complications have a significant impact on the survival of patients, who have received liver transplants because of the invasive surgical procedures involved and the need for immunosuppression and are closely related with in-hospital mortality.^1,7,8^ The preventive strategies of infectious complications after transplantation improve short-term outcomes after organ transplantation.

Nutritional support has been recognized as a vital component of the management of liver transplant recipients to help patient recovery. The advantages of enteral nutrition compared with parenteral nutrition as nutritional support for critically ill patients with respect to infectious complications are well recognized.^9^ European Society for Parenteral and Enteral Nutrition guidelines recommend early initiation of normal food intake or enteral feeding after organ transplantation as soon as possible.^3,10^

Several studies have examined the prevalence of posttransplantation bacterial sepsis in patients undergoing deceased donor liver transplantation (DDLT), and the benefits of perioperative nutritional therapies were proved in this setting.^4,11^ Many transplant centers in Korea have used living donors as a source for LT because of a limited number of available deceased donors. In contrast to DDLT, the evidence of the beneficial effects was little when living donor liver transplant (LDLT) recipients received early enteral nutrition.

In this study, we prospectively analyzed nutritional parameters in a group of patients undergoing early enteral feeding after LDLT and the relationship between enteral feeding and short-term clinical outcomes.

## MATERIALS AND METHODS

### Patients

Current study was designed a pilot randomized control trial to evaluate perioperative changes in nutritional parameters in the early posttransplant period after LDLT. The study included 36 consecutive patients who underwent elective LDLT at Samsung Medical Center from January 2013 to October 2013. The study was approved by the Samsung Medical Center's Institutional Review Board in Seoul. All participant patients provided written consent in the study. The patients were divided into 2 groups by using a block method for randomization: a “control” group (n = 19) and an “EN” group (n = 17). None of the patients were excluded from the analysis and all 36 patients were included in the per-protocol analysis.

### Assessment of Nutritional Status

Nutritional assessment was performed by experienced one dietician during thorough evaluation before LT. Body mass index (BMI) was calculated using body weight (kg)/height (m^2^). Body weight was measured before transplantation. Ideal body weight was computed by estimated weight. Mid-arm circumference (MAC, cm) was measured with a spring tape at the midpoint between the tip of the acromion and the ulnar process on the nondominant side of the arm hanging. Triceps skinfold measurement was measured in nondominant arm using a Harpender Skinfold Caliper. Midarm muscle circumference (MAMC) was calculated by the formula: MAMC = MAC – [π triseps skinfold thickness].^11^ The subjective global assessment (SGA) integrated using weight loss or gain, dietary history, gastrointestinal symptoms, medical history, coexisting medical conditions, physical activities, and physical signs of malnutrition of the patients. Malnourishment was defined as less than 5% of MAMC.

### Enteral Feeding

**FIGURE 1:**
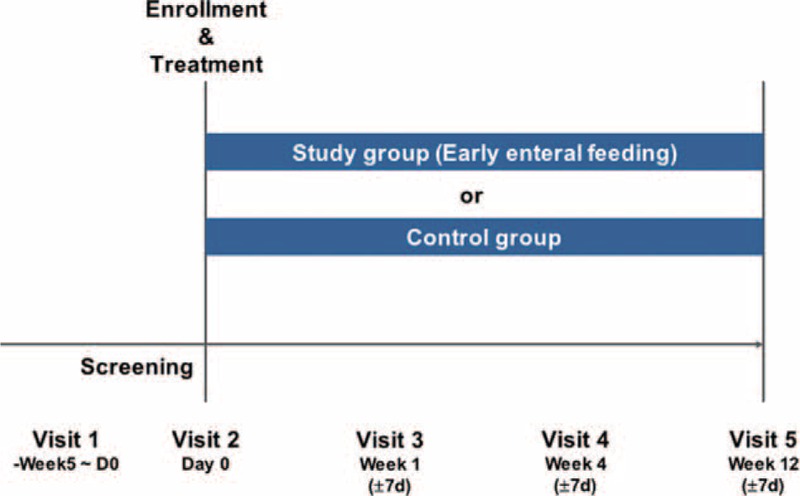
Study design.

Figure 1 shows the nutritional intervention schedule used in this prospective study. We provided enteral nutrition after LDLT via a nasogastric tube placed in the stomach several days after the operation. We routinely started enteral feeding within 12 hours of tube replacement for patients without enteral anastomosis. Enteral nutrition was started at 20 mL/hour for 12 hours and, if well tolerated, the enteral infusion rate was increased to 60 mL/hour by postoperative 5 days. A low residual enteral liquid diet (Mediwell RTH 500^®^, MDwell.Inc, Seoul, South Korea) was administered. Enteral feeding was discontinued once a patient could eat more than 50% of the provided regular diet.

### Antimicrobial Prophylaxis

Perioperative prophylaxis consisted of intravenous cefotaxime (4 g/day) and ampicillin sulbactam (6 g/day) given 4 times/day for 2 days after LDLT, and was started 30 minutes before the operation. If bacterial sepsis was clinically suspected, broad-spectrum antibiotics were administered empirically. Central venous catheters placed in the internal jugular vein were usually removed within 7 days after LDLT and replaced with a peripheral catheter.

### Outcomes

All outcomes were evaluated during the first 3 months after LDLT. The primary endpoint was occurrence of infectious complications and the secondary end points were total length of stay in the hospital, improvement in nutritional status, episodes of acute rejection, bile duct complications, graft failure, and mortality.

Bacterial, fungal, and cytomegalovirus (CMV) infections were continuously monitored after LDLT. Bacterial and fungal infections were diagnosed when they had clinical manifestations and causative organisms were isolated simultaneously. Cytomegalovirus infection was diagnosed as a CMV pp65 antigen-positive cell number greater than 1 positive cell per 200,000 white blood cells in whom CMV antigen was not detectable previously.^12^

The procedures used for biliary reconstruction in LDLT recipients and the prevention of infectious complications after LDLT were described previously.^13,14^ Bile duct complications were defined as biliary stricture or biliary leakage after LDLT.

### Statistical Analysis

Continuous variables were presented as the median and range. Data resulting from categorical variables are expressed as percentages or counts. Differences in continuous variables between the control and EN groups were analyzed by the Mann–Whitney *U* test. Differences in frequency were analyzed by the χ^2^ test or Fisher exact test. Sequential nutritional assessments between the 2 groups were evaluated using a mixed model test. Multivariate analyses were performed using the logistic regression model. All statistical analyses were performed with SPSS 21.0 software. All reported *P* values were 2-sided, and a *P* value <0.05 was considered statistically significant.

## RESULTS

### Patients

Clinical features of the subjects in both groups are shown in Table 1. The median age of the control group and EN group was 52 years (range: 36–64) and 52 years (range: 43–65), respectively. No significant differences in sex, BMI, or past history of hypertension and diabetes were noted between the control and EN groups. The 2 groups were comparable on the basis of diagnosis, Child-Pugh class, and model for end-stage liver disease score.

**TABLE 1:**
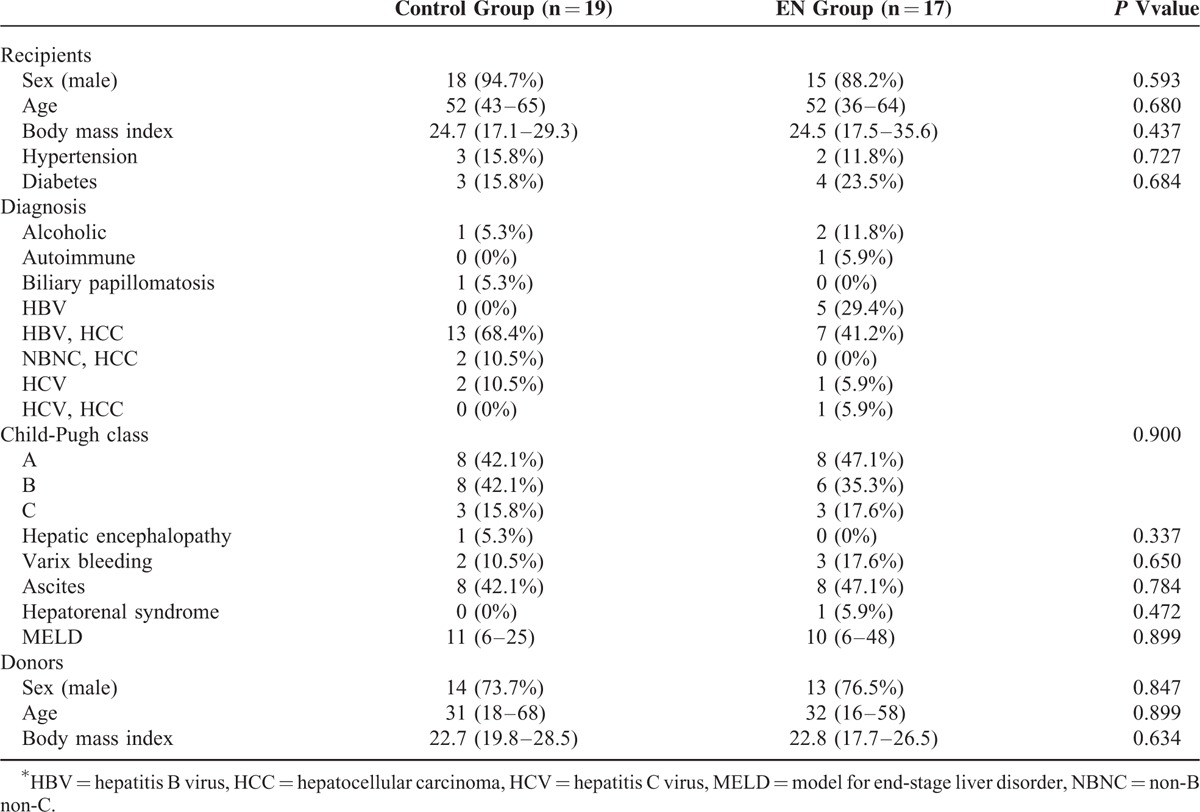
Baseline Characteristics

All patients received a right liver graft from a living donor and underwent duct-to-duct biliary anastomosis for reconstruction. There were no statistical differences in graft-to-recipient weight ratio, graft volume/standard liver volume, recipient operative times, donor operative times, cold ischemic time, warm ischemic time, steatosis, postoperative intensive care unit (ICU) stay, and hospitalization between the 2 groups (Table 2).

**TABLE 2:**
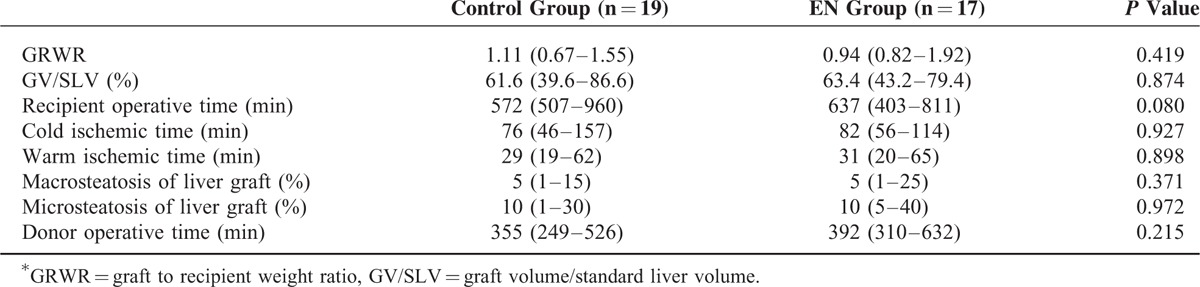
Perioperative Characteristics

Two patients in the EN group could not tolerate early enteral feeding; one had ileus and the other had vomiting. The remaining 15 patients who received early enteral feeding tolerated it well.

### Nutritional Changes Between Early Enteral Feeding and Control

**FIGURE 2:**
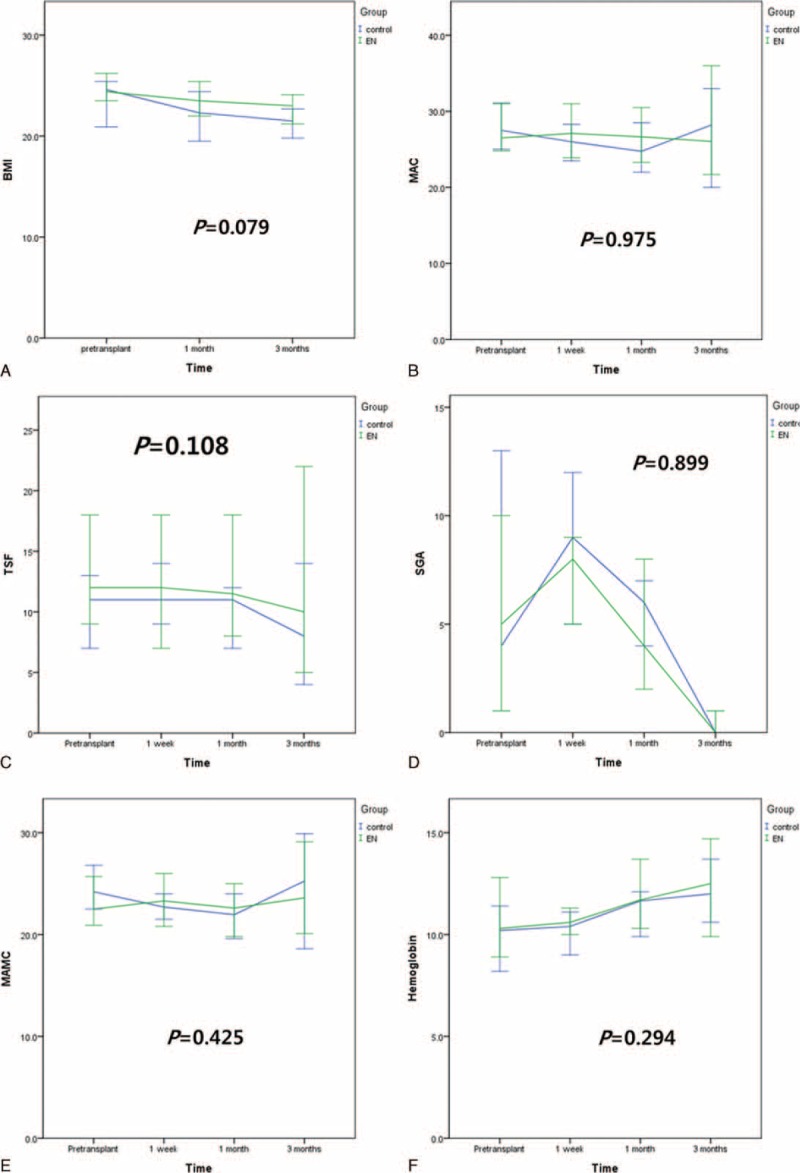
Nutritional changes after living donor liver transplantation. A, Body mass index. B, Mid-arm circumference. C, Triseps skinfold thickness. D, Subjective global assessment. E, Mid-arm muscle conference. F, Serum hemoglobin levels.

**FIGURE 3:**
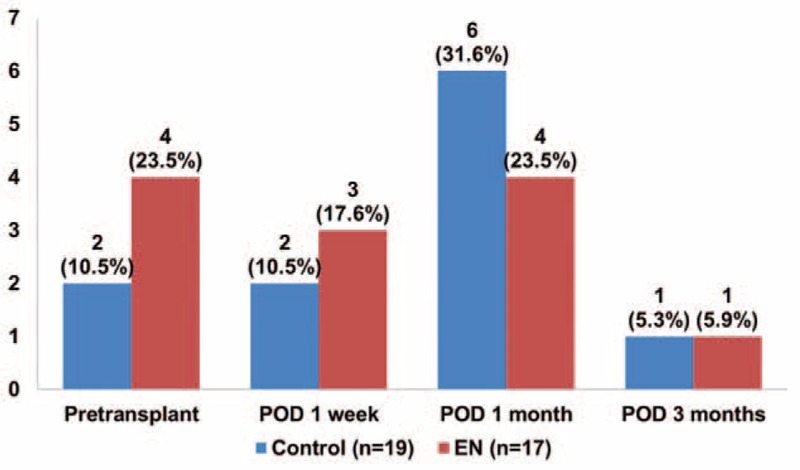
Proportion of malnourished patients (mid-arm muscle conference < 5%).

Nutritional status based on BMI, MAC, triseps skinfold thickness, subjective global assessment, and MAMC at pretransplant and postoperative 1 week, 1 month, and 3 months was not different between the EN group and control group (Fig. 2). The proportion of malnourished patients at pretransplant was 10.5% in the control group and 23.5% in the EN group. At 1 month after LDLT, the proportion of malnourished patients in the control group was higher than that in the EN group (31.6% versus 23.5%; Fig. 3) although the difference was not statistically significant. Hemoglobin levels at pretransplant and postoperative 1 week, 1 month, and 3 months were not different between the 2 groups. The median time to starting an oral diet in the EN group and control group was 4 days (range: 3–13 days) and 3 days (range: 3–8 days), respectively (*P* = 0.945).

### Clinical Outcomes

Early enteral feeding did not significantly influence ICU stay and hospitalization. Only 1 patient in the EN group developed biopsy-proven acute cellular rejection during the first 3 months. No significant difference in the incidence of biopsy-proven acute cellular rejection was noted between the 2 groups after LDLT. The incidence of bile duct complications in the control group was higher than that in the EN group (31.6% versus 5.9%, *P* = 0.041). Differences in the rates of infection between the 2 groups were detected. Bacterial infections occurred in 63.2% of the control group compared with 29.4% of the EN group (*P* = 0.043). Multivariate analysis revealed that early enteral feeding was closely associated with bacterial infection (odds ratio, 0.178; 95% confidence interval, 0.034–0.928; *P* = 0.041), but was not related to bile duct complications (odds ratio, 0.066; 95% confidence interval, 0.003–1.339; *P* = 0.077). No statistically significant differences existed between the 2 groups for CMV, fungal, and viral infection excluding CMV (Table 3). There were no cases of graft failure or in-hospital mortality.

**TABLE 3:**
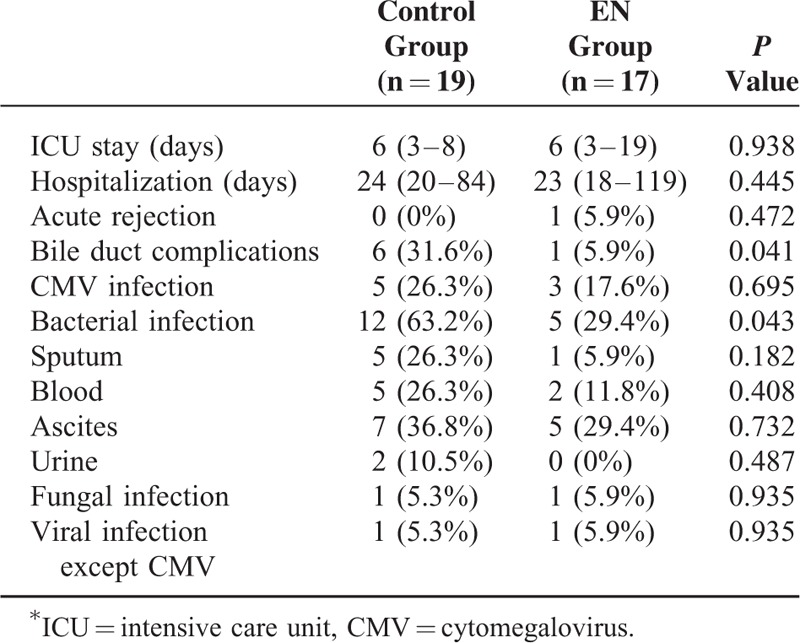
Outcomes at the First 3 Months after Living Donor Liver Transplantation

## DISCUSSION

Nutritional support provided immediately posttransplant facilitates nutritional and medical recovery in DDLT.^1,2^ Most patients undergoing LT have poor nutrition and are therefore good candidates for early enteral nutrition. The effects of postoperative enteral feeding in LDLT, however, have not been analyzed.

Compared with DDLT, LDLT involves smaller grafts and is scheduled as relatively elective surgery. The small size of graft in adult to adult LDLT has the risk of graft failure and patient death because of increased portal venous pressure, impaired bowel motility, bacterial translocation, ascites production, hyperbilirubinemia, and bleeding tendency by prolonged prothrombin time.^15^ These factors might increase the risk of bacterial sepsis in LDLT compared with DDLT.

In the current study, the incidences of bacterial infections or biliary complications in LDLT recipients who did not receive enteral feeding was significantly higher than that in the early EN group. Multivariate analysis reported that early enteral feeding was closely associated with the prevention of bacterial infections, but was not related to biliary complications. We discovered that early enteral nutrition also reduced the incidence of bacterial infection after LDLT. The period spent in ICU and the time of hospitalization after LDLT, however, were not influenced by early enteral feeding.

Infection is one of the most serious complications after liver transplantation. Bacterial infections in liver transplant recipients are associated with an increased mortality rate.^8^ The intestine contains the largest population of bacterial flora in the body, and both the intestinal immune system and mucosal barrier system play key roles in protecting against bacterial infection.^16^ Bacterial overgrowth and suppression of the intestinal antibacterial defense system are particular problems in patients with hepatic dysfunction, and are caused by portal hypertension resulting in intestinal edema and decreased peristalsis.^17^ Previous studies have indicated that sepsis is related to bacterial translocation and enterogenous endotoxemia, which results from intestinal mucosa barrier injury by total hepatic vascular exclusion and reperfusion.^8,18^ Enteral nutrition stimulates bile flow and portal blood flow, prevents intestinal mucosal atrophy, and preserves intestinal structure and function.^16^ In the current study, the overall proportion of patients with a bacterial infection was 63.2% in the control group and 29.4% in the EN group. In addition, trends toward a decrease in CMV infection were detected in the EN group.

A patient's nutritional status can worsen rapidly during the first 2 weeks postoperative as a result of preoperative malnutrition, surgical stress, immunosuppressive therapy, postinterventional complications, postoperative protein catabolism, and fasting.^19^ Thus, optimizing the nutrient intake over this period is critical to promote wound healing and hepatocyte recovery.^1,20^ The goal of nutrition therapy in the early posttransplant period is to ensure adequate protein and calorie provision to avoid protein breakdown.^5^

Cytokines play a major role in the inflammation. Therefore, the association between posttransplant complications and T-helper cytokines level has been studied to understand immune system modulation.^21^ Recent study reported that patients with biliary complications had higher interleukin (IL)-2, IL-4, and IL-12 levels than patients without. We suspected that early enteral feeding may affect the serum cytokine levels.^22^ Current study, however, did not include the relation between serum cytokine levels and biliary complications, so we could not draw a conclusion, which early enteral nutrition did not prevent biliary complications after LDLT.

Studies indicate that many patients are malnourished (30%–60%) at transplantation.^1,2^ A recent study reported that perioperative nutritional therapy improved survival in patients with low skeletal muscle mass, but not in patients with normal or high skeletal muscle mass.^23^ Severe malnutrition, however, was identified in only 16.7% (n = 6) of our patients because hepatocellular carcinoma was the main etiology for transplantation and LDLT does not involve a wait time. Therefore, graft failure and perioperative mortality were not observed in our series. As a result, we could not investigate the correlation between early enteral feeding and mortality. In addition, early enteral feeding did not significantly improve the nutritional status.

The current study had several limitations. First, our study did not compare calorie intake between the 2 groups. Second, the number of malnourished patients was low because of the nature of LDLT. Third, there was an inadequate selection bias because of the small number of patients. Fourth, the low number of events related to graft or patient survival could have obscured the effect of nutritional status on these parameters.

In conclusion, the current study encourages the use of early enteral feeding after LDLT. Early enteral feeding was well tolerated in most patients and resulted in a lower rate of bacterial infection and bile duct complications compared with patients who did not received early nutritional support.
